# Disparities exist between National food group recommendations and the dietary intakes of women

**DOI:** 10.1186/1472-6874-11-37

**Published:** 2011-08-08

**Authors:** Michelle L Blumfield, Alexis J Hure, Lesley K MacDonald-Wicks, Amanda J Patterson, Roger Smith, Clare E Collins

**Affiliations:** 1 School of Health Sciences, Faculty of Health, University of Newcastle, Callaghan, NSW, 2308, Australia; 2 School of Medicine and Public Health, Faculty of Health, University of Newcastle, Callaghan, NSW, 2308, Australia; 3 Mothers and Babies Research Centre, Hunter Medical Research Institute, John Hunter Hospital, Level 3, Endocrinology, Locked Bag 1, Hunter Region Mail Centre, NSW, 2310, Australia

## Abstract

**Background:**

Preconception and pregnancy dietary intakes can influence the health of future generations. In this study we compared the food intakes of reproductive-aged women by pregnancy status, to current Australian recommendations.

**Methods:**

Data are from the Australian Longitudinal Study on Women's Health, younger cohort aged 25-30 years in 2003, with self-reported status as pregnant (n = 606), trying to conceive (n = 454), given birth in the last 12 months (n = 829) or other (n = 5597). Diet was assessed using a validated 74-item food frequency questionnaire. Food group servings and nutrient intakes were compared to the Australian Guide to Healthy Eating (AGHE) and Australian Nutrient Reference Values (NRVs).

**Results:**

No women met all AGHE food group recommendations. Highest adherence rates [mean (95% CI) servings/day] were for meat [85%, 1.9(1.8-1.9)], fruit [44%, 2.1(2.1-2.2)] and dairy [35%, 1.8(1.8-1.9)], with < 14% meeting remaining recommendations. Women who achieved NRVs (folate, iron, calcium, zinc, fibre) for pregnancy, breastfeeding and adult life stages were 1.5%, 3.3% and 13.7%, respectively. Compared to AGHE, women consumed more servings of fruit (4.9 vs 4.0;*P *= 0.034) and dairy (3.4 vs 2.0;*P *= 0.006) to achieve pregnancy NRVs; more dairy (2.9 vs 2.0;*P *= 0.001), less fruit (3.9 vs 5.0;*P *< .001) and vegetables (3.4 vs 7.0;*P *< .001) to achieve breastfeeding NRVs; more fruit (3.6 vs 3.0;*P *< .001), dairy (2.5 vs 2.0;*P *< .001), meat (1.8 vs 1.5;*P *= 0.015), less vegetables (3.6 vs 5.0;*P *< .001) to achieve adult NRVs.

**Conclusions:**

The AGHE does not align with contemporary diets of Australian women or enable them to meet all NRVs. Current tools to guide food consumption by women during pregnancy require revision.

## Background

Dietary intake both prior to conception and during pregnancy can influence foetal, neonatal and longer-term health outcomes for mother and child [1-3]. Therefore, the nutritional status of reproductive-aged women impacts on the health of future generations [1].

The diets of women in developed countries are commonly found to be suboptimal, despite extensive strategies to improve diet quality [4-7]. In particular, maternal intakes have been characterised as being high in total energy, saturated fat and sodium, and low in dietary fibre, essential fatty acids, fruits and vegetables [7,8]. Women do not appear to consume a wide variety of nutritious foods thus contributing to an inadequate intake of several micronutrients important in pregnancy [8-10].

Preventative strategies that focus on improving diet quality through food-based approaches have been developed to guide individuals to improve dietary choices throughout the life cycle. Worldwide, food selection guides continue to have a strong focus on promoting the consumption of a wide variety of foods in order to achieve nutritional adequacy and are a commonly used National educational tool [11-14].

Data on whether reproductive-aged women consume foods in line with National food-based recommendations is limited. Studies have reported nutrient and food group intakes or grams of specific foods and/or food groups [6,10,15-18], but the adequacy of the whole diet relative to National recommendations has rarely been examined. Reporting grams of foods or food groups may be beneficial as it can facilitate international comparisons of dietary intakes, despite varying serving sizes in recommendations. However, this approach fails to evaluate the appropriateness of the relevant National recommendations in relation to population group intake.

In Australia limited data are available that describe the dietary intakes of reproductive-aged women [5,10,19,20]. Members of our team have recently shown in a representative sample of women from the Australian Longitudinal Study on Women's Health (ALSWH) that reproductive-aged women consume an inadequate variety of nutritious foods and commonly fail to meet key nutrient targets [10]. This was reported in terms of a diet quality score and nutrient intakes. It did not evaluate specific food intakes and eating patterns with National recommendations for women at this important life stage.

The aim of this study is to: (i) investigate whether young Australian women consume foods in accordance with National food group recommendations; (ii) determine if Australian women are achieving recommended intakes of folate, iron, calcium, zinc and fibre and (iii) if yes, to describe the respective food group intakes that are associated with optimal nutrient intakes. We hypothesise that young Australian women do not consume foods in accordance with National food recommendations, but may be able to achieve adequate intake of nutrients important for pregnancy through specific food patterns.

## Methods

### Data collection

Cross-sectional, self-reported data collected prospectively as part of the ALSWH were accessed. ALSWH recruited 41,512 women in three cohorts with baseline surveys issued in 1996 ('18-23 years', '45-50 years' and '70-75 years') [21]. Women were randomly selected from Australia's nationalised medical care system, Medicare, by the Health Insurance Commission who centrally administers this system. Women from rural areas were oversampled in attempt to gain a sample representative of the Australian population. Further details of the ALSWH have been published elsewhere [22-24]. This analysis includes only data from the younger cohort (n = 9076), who were aged 25 to 30 years at the time of completing the mailed Survey 3 food frequency questionnaire (FFQ) in March 2003. The follow-up rate at Survey 3 was 61.4% [23]. Ethical approval for the study was obtained from the Human Research Ethics Committee of the University of Newcastle.

The Dietary Questionnaire for Epidemiological Studies (DQES) version 2, a 74-item FFQ, was used and includes food and beverage data but does not report on vitamin or mineral supplement use. This FFQ reports usual intake for the previous 12 months and has been validated in a cohort of young Australian women [25].

### Sample

Details of the sample have been described [23,10]. Briefly, four groups were used to define pregnancy status: (i) pregnant (*n *606); (ii) trying to conceive (*n *454); (iii) given birth in the last 12 months (*n *829); and (iv) other (*n *5597) [10]. Subjects were excluded from these analyses if: (i) their pregnancy status could not be determined (*n *111); (ii) they could be grouped into more than one pregnancy category (*n *61); or (iii) their calculated energy intake was < 4.5 or > 20.0 MJ/d (*n *1418) [10,17]. A total of 7486 women were included in the analysis.

Detailed demographics have been reported elsewhere [10,23]. Table 1 reports the demographic characteristics of women in the ALSWH by pregnancy status. At both recruitment and Survey 3, the sample was broadly representative of the general population of Australian women the same age when compared to Australian census data, with a slightly higher representation of women who were married or in a *de facto *relationship and with post-school education [23]. The factors assessed were marital status, country of birth, education, housing situation and employment status [23].

**Table 1:** Demographic characteristics of women in the Australian Longitudinal Study on Women's Health by pregnancy status

	Trying to conceive (*n *454, 6.1%)		Pregnant (*n *606, 8.1%)		Birth < 12 mths ago (*n *829, 11.1%)		Other (*n *5597, 74.8%)	
	**Mean**	**95% CI**	**Mean**	**95% CI**	**Mean**	**95% CI**	**Mean**	**95% CI**
	
Age (years)	27.5*	27.4-27.6	27.4*	27.3-27.5	27.5*	27.4-27.6	27.1	27.1-27.1
Height (cm)	166.7	166.1-167.3	165.7	165.1-166.3	166.3	165.8-166.8	166.2	166.0-166.4
Weight (kg)	70.4	68.9-71.9	-	-	69.4*	68.4-70.4	67.1	66.7-67.5
	%		%		%		%	
	
Excluded based on energy < 4.5 or > 20.0 MJ/d	15.1		7.2*		6.7*		18.0	
Post-school education	68.2*		70.1*		65.6*		75.4	
Urban resident	67.7*		65.1*		60.0*		75.0	
Married/*de facto *status	96.7*		96.2*		93.9*		51.4	
Current smoker	22.7*		9.2*		17.1*		25.9	
Difficulty managing on available income	38.0		39.9		54.4*		38.4	
Inactive/low level physical activity	45.6*		68.9*		62.3*		40.9	
BMI (kg/m^2^)								
Underweight (< 18.5)	4.8		-		3.3		4.8	
Normal (18.5-24.99)	52.8*		-		53.1*		60.3	
Overweight (25-29.99)	19.9		-		26.9*		20.9	
Obese (≥ 30)	22.5*		-		16.8		14.0	

CI, Confidence Interval. BMI, Body mass index.

* Statistically significant difference (*P *< 0.05) compared with the total sample in the 'other' group.

Reproduced and adapted with permission from: Hure A, Young A, Smith R, Collins C: Diet and pregnancy status in Australian women. *Public Health Nutr *2009, 12: 853-61.

### Australian Guide to Healthy Eating

Australia's National food selection guide is the Australian Guide to Healthy Eating (AGHE) [26]. It was designed to encourage daily food consumption from each of five core food groups of breads/cereals (grains), lean meat and substitutes (including eggs, nuts and legumes), vegetables (including legumes), fruit and dairy, in proportions that are consistent with the Dietary Guidelines for Australians [27]. A non-core or 'extras' food group contains foods that do not belong to the core groups and are recommended to be consumed in limited amounts due to their energy density and/or minimal nutrient contribution. Recommended servings for each food group have been developed for different population subgroups based on meeting recommended nutrient values [28-30]. The AGHE recommended daily servings vary for non-pregnant, pregnant and breastfeeding women (Table 2).

**Table 2:** The Australian Guide to Healthy Eating daily energy and food group recommendations for women

	Energy (kJ)	Breads & Cereals	Fruit	Vegetables	Dairy	Meat & Alternatives	Extras
Adult 19-60 years	7200-11300	4-9	2	5	2	1	0-2.5
		4-6	2-3	4-7	2-3	1-1 1\2	0-2.5
Pregnant	8100-10900	4-6	4	5-6	2	1.5	0-2.5
Breastfeeding	9200-12300	5-7	5	7	2	2	0-2.5
Adult 60+ years	6500-9300	4-7	2	5	2	1	0-2.5
		3-5	2-3	4-6	2-3	1-1 1\2	0-2.5

Food group data is reported as the recommended range for number of serves per day^1,2^

^1 ^Defined by the Australian Guide to Healthy Eating: Pregnant intake averaged over 40 weeks, Breastfeeding intake during the first six months of breastfeeding. Reproduced and adapted with permission from: Smith A, Kellett E, Schmerlaib Y: *The Australian Guide to Healthy Eating: Background information for nutrition educators*. Canberra, Commonwealth of Australia; 1998.

^2 ^Serve size (FFQ categories) (a) Breads & Cereals: bread 60 g, cereal 40 g, cooked porridge 230 g, muesli 65 g, cooked rice/pasta/noodles (including lasagne) 180 g, dry biscuits 40 g (b) Fruit: fruit whole (including canned fruit) 150 g, fruit juice 125 ml (c) Vegetables: vegetable whole (including potatoes cooked without fat) 75 g; avocado 30, lettuce/endive/salad greens 36 g, tomato sauce/paste 20 g (d) Dairy: milk 250 ml, cheese 40 g, yoghurt 200 g, flavoured milk 250 ml (e) Meat & Alternatives: beef/veal/chicken/lamb/pork 85 g, fish (steamed/grilled/baked/canned) 100 g, ham 100 g, baked beans/tofu/soy beans/soy bean curd/other beans (including chickpeas, lentils etc) 80 g, nuts 40 g, eggs 100 g (f) Extras: sweet biscuit 35 g, cakes/sweet pies/tarts/other sweet pastries 40 g, meat pies/pasties/quiche/other savoury pies 60 g, pizza 60 g, hamburger 60 g, chocolate 25 g, peanut butter 25 g, potato crisps/corn chips/Twisties^® ^30 g, jam/marmalade/honey/syrups 45 g, Vegemite^®^/Marmite^®^/Promite^® ^100 g, ice-cream 50 g, bacon 50 g, corned beef/luncheon meats/salami 110 g, sausages/frankfurters 55 g, fried fish 65 g, fat spread 20 g, sugar 40 g, fries 60 g, light beer 600 ml, heavy beer 400 ml, wine (including sparkling wines) 200 ml, spirits/liqueurs 60 ml, fortified wines/port/sherry 60 ml.

### Nutrient Reference Values

The National Health and Medical Research Council of Australia nutrient reference values (NRVs) prescribe specific daily nutrient intake targets to optimise health and/or avoidance of nutritional deficiency [31]. The most appropriate NRVs for comparison with population group intakes are estimated average requirements (EAR) and adequate intakes (AI) [31]. The EAR is the daily nutrient level estimated to meet the requirements of half the healthy individuals in a particular life stage and gender group and is used to estimate the prevalence of inadequate intakes, with the proportion below the EAR providing a suitable approximation of the prevalence of inadequacy [31]. When an EAR is not able to be set an AI, which is the average daily nutrient intake level that is assumed to be adequate, based on observed or experimentally determined nutrient intake estimates by a group of apparently healthy people, is used instead [31].

### Statistical Analysis

To improve the validity of the dietary analyses, the energy cut off values recommended by Meltzer et al. (2008) were applied across all pregnancy groups by excluding those who reported daily energy intakes < 4.5 or > 20.0 MJ/d [17]. Basal metabolic rate could not be used to estimate those most likely to have misreported intake because weight data was not reported for pregnant populations.

Main outcome measures included food group intake in grams, daily servings and the proportion meeting AGHE recommendations. Macronutrient intakes and daily food group servings were also examined for those meeting the EAR for folate, iron, calcium, zinc and AI for fibre. These five nutrients were chosen because their role in optimizing reproductive health has been studied extensively [32,33].

Food servings per day were calculated using portion sizes described in the AGHE [26]. When portion sizes were not specified standard portions were derived using NUTTAB 2006, the most recent National government food composition database of Australian foods [34], and the energy value attributed to each AGHE food group [26]. The portion sizes for each FFQ category used to calculate each food group serving size can be found in Table 2. Comparisons were made with the minimum number of AGHE servings for women aged 19 to 60 years [26].

Data were tested for normality. Normally distributed data reported as mean (95% CI) and not normally distributed data reported as median [IQR]. Multiple comparisons were performed using two-sample t-tests or the Kruskal-Wallis test for continuous data and the chi-squared statistic for categorical data.

To determine whether Australian women can achieve adequate intakes of folate, iron, calcium, zinc and fibre, dietary profiles were calculated using the EAR or AI for each life stage (i.e. adult 19-60 years, pregnancy and breastfeeding). All data manipulation and statistical analyses were performed using Intercooled Stata 11.0 (Stata, College Station, Texas, USA). *P*-values < 0.05 were considered statistically significant.

## Results

The AGHE food group recommendations for women are presented in Table 2. Table 3 reports the daily food group intakes in grams, daily servings and as the percentage of women achieving the minimum number of daily AGHE food group servings.

**Table 3:** Daily food group consumption and the percentage of women achieving daily food group recommendations for adults 19-60 years in the Australian Guide to Healthy Eating, for the young cohort of the Australian Longitudinal Study on Women's Health by pregnancy status

	Trying to conceive (*n *454)		Pregnant (*n *606)		Birth < 12 mths ago (*n *829)		Other (*n *5597)		All (*n *7486)	
	**Median**	**IQR**	**Median**	**IQR**	**Median**	**IQR**	**Median**	**IQR**	**Median**	**IQR**
	
**Food Group Intakes**										
Breads/cereals (g/d)	194	149-264	202	154-267	213***	158-282	201	150-272	202	151-273
Fruit (g/d)	245	143-383	308***	188-485	258	141-393	248	140-383	253	144-391
Vegetables (g/d)	164	125-219	154	119-214	179***	135-236	159	116-216	161	119-218
Dairy (g/d)	383	232-439	407***	287-511	393***	237-457	360	227-424	379	229-435
Meat/alternatives (g/d)	147	105-200	135	96-188	156***	114-206	142	103-197	143	104-198
Extras (g/d)	133	92-183	139	96-193	156***	110-214	130	93-183	133	95-188

**Food Group Servings**										
Breads/cereals (servings/d)	2.5	1.8-3.2	2.6***	2.0-3.5	2.7***	2.1-3.6	2.4	1.8-3.1	2.5	1.9-3.2
Fruit (servings/d)	1.8	1.0-2.7	2.2***	1.3-3.5	1.9	1.0-2.8	1.8	1.0-2.7	1.8	1.0-2.8
Vegetables (servings/d)	2.2	1.6-2.9	2.1	1.6-3.0	2.4***	1.8-3.2	2.1	1.5-2.9	2.2	1.6-3.0
Dairy (servings/d)	1.8	1.3-2.3	2.0***	1.6-2.6	1.9***	1.4-2.3	1.7	1.2-2.2	1.7	1.2-2.2
Meat/alternatives (servings/d)	1.6	1.1-2.1	1.5	1.1-2.0	1.7***	1.2-2.2	1.5	1.1-2.1	1.5	1.1-2.1
Extras (servings/d)	4.1	2.9-5.4	4.1	3.0-5.7	4.5***	3.3-6.2	4.2	3.1-5.8	4.2	3.1-5.8

	Mean	95% CI	Mean	95% CI	Mean	95% CI	Mean	95% CI	Mean	95% CI
	
**Meeting Recommendations^1^**										
Breads/cereals (%)	11.9	9.1-15.2	14.0**	11.4-17.0	15.7***	13.3-18.3	10.3	9.5-11.1	11.3	10.6-12.0
Fruit (%)	41.4	36.8-46.1	55.4***	51.4-59.5	45.4	41.9-48.8	42.9	41.6-44.2	44.1	43.0-45.2
Vegetables (%)	13.0	10.0-16.4	10.9	8.5-13.6	13.5	11.3-16.0	11.5	10.7-12.4	11.8	11.0-12.5
Dairy (%)	36.3	31.9-41.0	50.3***	46.3-54.4	44.4***	41.0-47.8	32.4	31.1-33.6	35.4	34.3-36.5
Meat/alternatives (%)	89.0**	85.7-91.7	82.3	79.1-85.3	87.5*	85.0-89.6	84.3	83.3-85.2	84.7	83.9-85.6
Extras (%)	16.1	12.8-19.8	14.9	12.1-17.9	10.9*	8.8-13.2	13.8	12.9-14.7	13.7	12.9-14.5

**Other**										
Daily energy intake (kJ)	7487	7269-7705	7929***	7729-8130	8277***	8099-8455	7367	7309-7428	7521	7467-7575
Total food weight (g)	1393	1355-1431	1519***	1482-1556	1480***	1450-1510	1365	1354-1375	1392	1382-1401
Energy density (kJ/g)	5.4	5.4-5.5	5.3***	5.2-5.4	5.7***	5.6-5.7	5.5	5.4-5.5	5.5	5.4-5.5

CI, Confidence Interval. Statistically significant difference compared with the 'other' group: *P < 0.05, ** *P *< 0.01, *** *P *< 0.001.

1 Defined by the Australian Guide to Healthy Eating food group recommendations for women aged 19-60 years. Smith A, Kellett E and Schmerlaib Y (1998). *The Australian Guide to Healthy Eating: Background information for nutrition educators*. Canberra, Commonwealth of Australia.

None of the women in any pregnancy group achieved the AGHE recommendations for all food groups. Highest adherence rates [proportion, mean (95% CI) servings/day] were for meat [85%, 1.9 (1.8-1.9)], fruit [44%, 2.1 (2.1-2.2)] and dairy [35%, 1.8 (1.8-1.9)], while less than 14% met the remaining food group recommendations (Table 3). The median [IQR] consumption of non-core, 'extra' foods was 4.2 [3.1-5.8] servings/day for all women, 60% greater than the recommended maximum of 2.5 servings/day (Table 3).

Women 'trying to conceive' had similar food consumption patterns to women in the 'other' group (Table 3). 'Pregnant' women reported a greater median daily intake of both fruit (308 g/d vs 248 g/d; *P *= < .001) and dairy (407 g/d vs 360 g/d; *P *= < .001) food groups compared to 'other' women (Table 3). This equates to an additional half serving of fruit and third serving of dairy. Despite the higher fruit intake, 82% of pregnant women failed to meet the AGHE fruit recommendations for pregnancy (Table 2). Women who had 'given birth in the last 12 months' reported greater median daily servings of breads and cereals (2.7 vs 2.4; *P *= < .001), vegetables (2.4 vs 2.1; *P *= < .001), dairy (1.9 vs 1.7; *P *= < .001), meat (1.7 vs 1.5; *P*< .001) and extras (4.5 vs 4.2; *P *< .001) compared to 'other' women (Table 3).

Table 4 reports the food group and macronutrient profile of women who met the EAR for folate, iron, calcium, zinc and AI for fibre for each female life stage (adult 19-60 years, pregnancy and breastfeeding) from food alone.

**Table 4:** Food group and macronutrient intake of women in the young cohort of the Australian Longitudinal Study on Women's Health, who met the Nutrient Reference Values for folate, iron, calcium, zinc and fibre, recommended for each life stage based on dietary intake only^1^

	Adults 19-60 years				Pregnancy				Breastfeeding			
	
Diet Type^2^	High-Extras Diet (*n *957)		Optimal Diet (*n *67)		High-Extras Diet (*n *105)		Optimal Diet (*n *6)		High-Extras Diet (*n *234)		Optimal Diet (*n *16)	
	***n***		***n***		***n***		***n***		***n***		***n***	
	
**Pregnancy Group**												
Trying to conceive	58		2		4		-		12		-	
Pregnant	108		11		12		-		32		4	
Birth < 12 months ago	154		5		24		1		45		2	
Other	637		49		65		5		145		10	

	Median	IQR	Median	IQR	Median	IQR	Median	IQR	Median	IQR	Median	IQR
	
**Food groups**												
Breads/cereals (servings/d)	4.0	3.3-4.9	4.2	3.2-4.9	5.9	4.8-7.2	6.7	4.9-8.3	5.0	4.2-6.2	5.2	4.0-6.5
Fruit (servings/d)	3.1	2.1-4.4	3.6	2.3-4.9	3.5	2.2-5.6	4.9	4.2-5.8	3.9	2.4-5.6	3.9	2.4-5.4
Vegetables (servings/d)	3.2	2.4-4.2	3.6	2.8-4.5	3.8	2.5-4.9	4.2	3.5-4.9	3.6	2.6-4.6	3.4	3.2-4.8
Dairy (servings/d)	2.2	1.8-2.9	2.5**	2.1-3.0	2.4	1.8-3.2	3.4**	2.9-5.3	2.4	1.8-3.1	2.9*	2.3-3.8
Meat/alternatives (servings/d)	2.2	1.6-3.1	1.8***	1.2-2.5	3.1	1.8-4.4	2.4	1.6-3.4	2.6	1.8-3.7	1.9*	1.3-2.9
Extras (servings/d)	5.9	4.1-8.2	1.8***	1.2-2.2	7.1	5.7-10.7	1.1***	0.6-1.7	6.8	4.9-9.6	1.8***	0.6-2.3

**Macronutrients**												
Energy (MJ)	10.6	9.0-12.6	7.7***	6.8-8.7	14.3	13.7-14.8	11.1**	8.8-13.3	13.0	12.6-13.4	9.5***	8.3-10.7
% total fat	35.1	31.2-38.5	26.2***	22.8-30.2	34.1	31.2-37.8	23.5***	20.3-29.3	34.3	30.3-38.2	26.0***	20.8-29.4
% saturated fat	14.4	12.1-16.6	9.7***	7.6-11.9	14.5	12.4-16.4	7.8**	5.8-8.9	14.1	11.9-16.3	8.8***	7.1-10.0
% monounsaturated fat	12.3	10.9-12.6	9.1***	7.6-10.8	12.0	10.7-13.5	8.2**	6.5-9.7	12.0	10.7-13.5	9.2***	6.7-10.8
% polyunsaturated fat	4.7	3.8-5.9	4.1***	3.0-5.5	4.5	3.7-5.4	4.0	3.1-10.1	4.6	3.6-5.8	4.2	3.2-6.4
% carbohydrate	43.4	39.8-46.9	49.2***	44.1-54.1	45.1	40.3-48.9	52.2**	48.9-56.9	44.3	40.8-48.1	49.5***	44.7-54.8
% protein	19.7	17.8-21.6	21.4***	19.4-24.0	19.1	17.2-22.0	21.1	18.9-23.9	19.6	17.6-21.6	22.6**	19.0-23.9

CI, Confidence Interval. Statistically significant difference compared with the 'High-Extras Diet' of the same life stage: *P < 0 < 05, **P < 0 < 01, ***P < 0 < 001.

^1 ^Nutrient Reference Values (a) Adults: Folate = 320 μg, Iron = 8 mg, Calcium = 840 mg, Zinc = 6.5 mg, Fibre = 25 g (b) Pregnancy: Folate = 520 μg, Iron = 22 mg, Calcium = 840 mg, Zinc = 9 mg, Fibre = 28 g (c) Breastfeeding: Folate = 450 μg, Iron = 6.5 mg, Calcium = 840 mg, Zinc = 10 mg, Fibre = 30 g. National Health and Medical Research Council: *Nutrient Reference Values for Australia and New Zealand*. Canberra, Commonwealth of Australia; 2006.

^2 ^High-Extras Diet, refers to women who achieved the EAR for folate, iron, calcium, zinc and AI for fibre AND consumed high non-core, 'extra' foods that contributed to macronutrient profiles for energy and saturated fat intakes above dietary recommendations; Optimal Diet, refers to the women who achieved the EAR for folate, iron, calcium, zinc and AI for fibre AND consumed less than 2.5 'extra' foods per day.

Two distinct dietary profiles were identified those who achieved the NRVs (EAR for folate, iron, calcium, zinc and AI for fibre): 'High-Extras diet' and 'Optimal diet' (Table 4). 'High-Extras diet' women achieved the selected NRVs (EAR for folate, iron, calcium, zinc and AI for fibre), but also consumed a high number of non-core, 'extra' foods (5.9-7.1 servings/day) with total energy and saturated fat intakes above current recommendations. The second dietary profile, 'Optimal diet', contained women who achieved the selected NRVs (EAR for folate, iron, calcium, zinc and AI for fibre) with optimal macronutrient intakes.

The number of women, from each pregnancy group, who consumed the 'High-Extras diet' or 'Optimal diet' for each life stage is reported in Table 4. Interestingly, no women (n = 0) from the pregnancy group reported an 'Optimal diet' for pregnancy, and only two women who reported trying to conceive consumed an 'Optimal diet' for adults 19-60 years.

When compared to the 'High-Extras diet', women who followed the 'Optimal diet' consumed less energy, total fat and saturated fat, but greater servings from the breads/cereals, fruit, vegetables and dairy food groups. Less than 1% of women were able to achieve recommended nutrient intakes following the 'Optimal diet' and they were more likely to have post-school qualifications (*P *= 0.029), not be in a married/*de facto *relationship (*P *= 0.022), be a non-smoker (*P *= 0.003) and to participate in a moderate to high level of physical activity, compared to the whole cohort population (*P *= 0.001).

When the 'Optimal diet' for each life stage was compared to AGHE recommendations, pregnancy NRVs (EAR for folate, iron, calcium, zinc and AI for fibre) were achieved by consuming greater daily servings of fruit (4.9 vs 4.0; *P *= 0.034) and dairy (3.4 vs 2.0; *P *= 0.006), breastfeeding NRVs (EAR for folate, iron, calcium, zinc and AI for fibre) were achieved by greater daily servings of dairy (2.9 vs 2.0; *P *= 0.001), lower servings of fruit (3.9 vs 5.0; *P *< .001), and vegetables (3.4 vs 7.0; *P *< .001), and adult NRVs (EAR for folate, iron, calcium, zinc and AI for fibre) by greater servings of fruit (3.6 vs 3.0; *P *< .001), dairy (2.5 vs 2.0; *P *< .001) and meat (1.8 vs 1.5; *P *= 0.015), lower servings of vegetables (3.6 vs 5.0; *P *< .001).

When the 'Optimal diet' was compared to the 'High-Extras diet', women following the 'Optimal diet' for pregnancy consumed greater servings of dairy (3.4 vs 2.4; *P *= 0.006). To achieve breastfeeding recommendations, women consumed greater servings of dairy (2.9 vs 2.4, P = 0.022) and lower servings of meat (1.9 vs 2.6, *P *= 0.017). Similarly, this effect was seen for those (n = 67; 0.9%) achieving the adult recommendations (dairy 2.5 vs 2.2, *P *= 0.004; meat 1.8 vs 2.2, *P *< .001).

## Discussion

This study provides insight into the food intakes of a population-based cohort of women defined by life stage and compares intakes to recommendations given in a National food selection guide. Most Australian women did not consume foods in accordance with the AGHE and this was associated with suboptimal nutrient intakes. However, women who did achieve the EAR or AI for key pregnancy nutrients (folate, iron, calcium, zinc and fibre) had eating patterns that differed slightly from AGHE recommendations. Greater consumption of fruit (4.9 vs 4.0 servings) and dairy (3.4 vs 2.0 servings) foods to meet nutrient recommendations for pregnancy, greater dairy (2.9 vs 2.0 servings) but lower fruit (3.9 vs 5.0 servings) and vegetables (3.4 vs 7.0 servings) to meet nutrient recommendations for breastfeeding and greater fruit (3.6 vs 3.0 servings), dairy (2.5 vs 2.0 servings) and meat intakes (1.8 vs 1.5 servings) but lower vegetables (3.6 vs 5.0 servings) to achieve nutrient recommendations for adults.

There was no evidence that women 'trying to conceive' consume greater intakes of nutrient rich foods prior to conception. This is concerning given that unplanned pregnancies are common [35] and the emerging evidence that nutrient intakes may exert an influence on foetal programming from the earliest stages of conception [1,2].

Importantly, we have shown that women can achieve the recommended intake of key nutrients required for childbearing through food intake alone, but through consumption patterns of core foods that differ from current AGHE recommendations. Potential reasons contributing to food group intake differences include: (i) Australian recommended nutrient intakes have been revised since the AGHE was published and (ii) disparity between contemporary eating patterns of reproductive-aged women and the AGHE. Current NRVs recommend higher intakes of folate, iron and calcium, and a lower intake of zinc compared to the nutrient recommendations that were modelled for women when the AGHE was developed [29,31]. A major limitation of the AGHE is that its food consumption recommendations do not meet the current EAR for iron during pregnancy [26]. While the AGHE is currently under revision, it may explain why pregnant women who met the EAR for folate, iron, calcium, zinc and AI for fibre reported consuming an extra serving of meat compared to AGHE recommendations (2.4 vs 1.5). When the AGHE was originally developed in 1998, no comprehensive National data was available on the actual food and nutrient intakes of pregnant and breastfeeding women. This study provides the first comparison of AGHE recommendations to actual dietary intake patterns of women and will be important data to inform future revisions.

Women following the 'Optimal diet' reached the specific NRVs (EAR for folate, iron, calcium, zinc and AI for fibre) for each life stage without consuming excess 'extra' foods. A higher consumption of 'extra' foods increases intakes of kilojoules, total fat, saturated fat and added sugars, while providing relatively few micronutrients [36,37]. This pattern of excess 'extra' foods applies to the majority of women in this sample. Results indicate that while nutrient intake appears correlated with energy intake, 'extra' foods displace the nutrient dense core foods which is clearly evident when we compare women following the 'Optimal diet' versus the 'High-Extras diet'.

While the criteria for the 'Optimal diet' were based on five nutrients (folate, iron, calcium, zinc and fibre), the dietary intake of women who followed an 'Optimal diet' also achieved the NRVs (both EAR and recommended dietary intakes) for all additional nutrients considered in the development of the AGHE (vitamins A, B1, B2, B3, B12, and C, magnesium, sodium and potassium), without exceeding upper limits [26,31]. This confirms that the 'Optimal diet' represents a nutritionally adequate diet and basing dietary recommendations on the food group intake of women who followed the 'Optimal diet' may help to optimize nutrient intake profiles within energy requirements. This can be used to inform future revisions of the AGHE and other food selection guides from Western countries, using contemporary food and nutrient intake data from women as opposed to modelling theoretical food patterns.

Our findings are similar to other nationally representative studies that have compared the diets of reproductive-aged women to National food group recommendations [4,6,16]. In the United States, the dietary intake of women aged 20-29 years (n = 675) were compared to the Food Guide Pyramid [4]. In comparison, fewer Australian women in our sample achieved food group recommendations for breads and cereals (11% vs 41%), vegetables, (24% vs 43%) and dairy (35% vs 56%) [4]. In Ireland, the dietary habits of women aged 18-65+ years (n = 5995) were compared to the Irish Food Pyramid [6]. Less than 1% met all food group recommendations and 10% did not meet any recommendations [6]. In comparison, fewer Australian women achieved food group recommendations for breads and cereals (11% vs 25%) and fruit and vegetables (21% vs 71%) [6]. Similar food group intakes were also reported in an Irish cohort of pregnant women (n = 1124) compared to the previously described Irish National sample [6,16]. Despite true population differences in National eating patterns between Australia, United States and Ireland, it is evident that a large proportion of reproductive-aged women internationally fail to meet National food group recommendations.

Greater diversity in food group recommendations that align with contemporary eating patterns of reproductive-aged women are urgently needed to optimize nutrient intakes during pregnancy and lactation. These should be incorporated into revised food selection guides to ensure this alignment provides greatest flexibility in food choices to accommodate a large number of different eating behaviours. Results confirm that Australian women prefer a varied diet, as opposed to a cereal based diet, while a combined fruit and vegetable recommendation could also be considered. A combined fruit and vegetable recommendation is advised by the United Kingdom and Ireland in the Eatwell Plate and the Irish Food Pyramid. Research into the effectiveness of a combined fruit and vegetable recommendation is necessary to ensure the nutrients supplied from each food group are optimized and to determine if this does encourage more women to consume an adequate intake of nutrients important in this life stage.

Limitations include that ALSWH did not report stage of gestation or weights in pregnant women. The DQES asks participants to report usual intake for the previous 12 months. While the DQES has been validated in a cohort of young Australian women [25], it has not been validated in all categories of women defined in this study. Past dietary recall is known to be influenced by current intake [38]. While the inexact alignment of the dietary reference period with the definition of some of the groups may explain some of the lack of difference between groups, statistically significant differences between groups were found and it does not account for the suboptimal intakes across the entire cohort. Furthermore, the ability of the DQES to report usual intake in this sample is strengthened by the similarities in the daily mean energy intake reported by our representative sample (7521 kJ) and the mean daily energy intake reported by women 25-44 years in the Australian 1995 National Nutrition Survey (7875 kJ/day) [5]. The ALSWH is the only large, nationally representative sample of young women in Australia and thus represents the best available population level data at this time.

No information was collected on vitamin supplementation however the purpose was to focus on nutrients supplied from food only. Findings are strengthened due to the large representative sample using a validated FFQ specifically designed for use in the Australian population. Results are comparable to international populations with similar demographic characteristics to this Australian sample.

## Conclusions

Eating patterns in young Australian women do not align with current National food selection guides. Women who achieve the NRVs for key nutrients important for childbearing (EAR for folate, iron, calcium, zinc and AI for fibre) generally consume more fruit and dairy compared to current recommendations. While young women can achieve a healthy diet through food intake, contemporary dietary patterns differ from Australian food selection guide recommendations. Given the importance of diet in reproductive-aged women, the appropriateness of recommendations made in international food selection guides needs to be evaluated and future revisions should consider providing alternative food group recommendations, to accommodate differing dietary patterns.

## Competing interests

The authors declare that they have no competing interests. MLB is supported by a PhD scholarship provided by the University of Newcastle and Newcastle Permanent Charitable Foundation, New South Wales, Australia. CEC is supported by a National Health and Medical Research Council Career Development Award.

## Authors' contributions

CEC, LKMW, and AJH helped design the study and directed its implementation, including quality assurance and control. AJP and RS provided ongoing input into the implementation, quality assurance and control aspects of the project. MLB helped design the study, worked on the statistical analyses and was responsible for the project's implementation including the preparation of the manuscript. All authors have made a significant contribution to the research and the development of the manuscript. All authors have read and approved the final manuscript.

## Pre-publication history

The pre-publication history for this paper can be accessed here:

http://www.biomedcentral.com/1472-6874/11/37/prepub
